# African Primary Care Research: Reviewing the literature

**DOI:** 10.4102/phcfm.v6i1.584

**Published:** 2014-02-25

**Authors:** Andrew Ross, Bob Mash

**Affiliations:** 1 Department of Family Medicine, Nelson R. Mandela School of Medicine, University of KwaZulu-Natal, South Africa; 2 Family Medicine and Primary Care, Stellenbosch University, South Africa

## Abstract

This is the second article in the series on African primary care research. The article focuses on how to search for relevant evidence in the published literature that can be used in the development of a research proposal. The article addresses the style of writing required and the nature of the arguments for the social and scientific value of the proposed study, as well as the use of literature in conceptual frameworks and in the methods. Finally, the article looks at how to keep track of the literature used and to reference it appropriately.

## Abstrait

**La recherche sur les soins de santé primaire en Afrique: Étude documentaire**. Cet article est le deuxième de la série consacrée à la recherche sur les soins de santé primaire en Afrique. Il se penche sur la marche à suivre pour rechercher des faits probants pertinents dans la littérature publiée qui puissent être utilisés dans l’élaboration d'une proposition de recherche. L'article traite du style d’écrit requis et de la nature des arguments en faveur de la valeur sociale et scientifique de l’étude proposée, ainsi que de l'utilisation de la littérature dans les cadres conceptuels et les méthodes. Enfin, l'article se penche sur la marche à suivre pour se tenir informé de la littérature utilisée et y faire référence de manière appropriée.

## Introduction

The previous article introduced the topic of primary care research in an African context.^1^ The various types of research were outlined and guidelines on choosing a topic were suggested. Before any research can be conducted, a proposal has to be approved by an institutional research and ethics committee. Headings for the development of a proposal were outlined in the previous article. It is helpful to use these headings as a formula. If you go step by step, from heading to heading, you will cover all the important aspects of the proposal and will usually have no problem with your institutional research and ethics committee. This second article in the series focuses on how to obtain and use the literature in preparing your proposal.

Before beginning to look for relevant literature, it is important to ensure that you are reasonably clear about your research question, aim and objectives. What is it that you are studying? What are you trying to investigate? Keep this at the front of your mind at all times. It is possible to read extensively and become overwhelmed by all the information. It is also possible to use too much background information and not focus clearly enough on your specific research question. Remember that you are not expected to read everything that has been written on the topic, but you should craft your arguments using the most relevant and important evidence.

Begin by reading generally around the topic. What have others said? As you familiarise yourself with the work that has been done previously on your topic you may start to focus or even redefine your research question. For example, you may become clearer as to what the gap in knowledge really is and what unanswered questions have been identified in the existing literature.

Hart^2^ suggested that the purpose of a literature review should be to:

- establish the context of the current study
- rationalise the significance of the study
- synthesise and gain new insights
- distinguish what has been done from what needs to be done
- discover important variables relevant to the current study
- relate ideas and theory to applications
- identify methodologies that have been used.

## Searching for the literature

Useful information will usually come from scholarly journals, books, internet sources and even unpublished theses. Articles from peer-reviewed journals are more likely to be methodologically sound than those from non-peer-reviewed journals. Try to find recent review articles, which already summarise the research on the topic of interest. Relevant systematic reviews that synthesise the evidence on a specific question should also be looked for. The Cochrane Library is a particularly useful source of systematic reviews. Try to include references from high impact journals, but also remember that African research is not always found in such European and American journals. You may need to conduct a specific search for African literature. For example, SABINET has an Index of South African Periodicals (ISAP) and a Union Catalogue of Theses and Dissertations (UCTD). EBSCOHOST also has Africa Wide NIPAD looking at African scholarship.

Broadly speaking, there are three entry points to the forest of scholarship. The most common is to use key words within search engines and databases so as to identify relevant and appropriate readings for your study. Useful search engines include Medline, Pubmed, EBSCO, Science Direct and Google scholar.

A second strategy, however, is to identify a few of the key scholars and their critical publications in your field of interest and to then follow their later publications and see who else is citing and using their work. Many search engines link you to work by the same author as well as other work that cites the article. Examining the references used by critical publications may also identify other key scholars and literature.

The third strategy is to locate the key journals or places where scholars publish in your field of interest and search specifically these journals for additional work that is relevant. In the discipline of family medicine, do not forget to look at our regional journals such as the African Primary Health Care and Family Medicine Journal or the South African Family Practice Journal.

It is important to use current literature. The bulk of your literature should be from the last five years and, ideally, none of your references should be more than 10 years old. The only exception is historical research which, for obvious reasons, traces patterns of scholarship and practice over time.

Be systematic as you search for relevant literature. Remember that you want to perform a smart, targeted search. As you locate the relevant articles, read the abstracts, assess their importance and relatedness to your work (discard any that you feel are not useful or related). If the abstract seems relevant, access and read the whole article.

## Style of writing

The style of writing should go beyond just summarising the literature (i.e. listing the studies you have read in a series of paragraphs), or even synthesising the literature (i.e. grouping the studies chronologically or by topic), to a critical appraisal of the literature (i.e. interpreting the quality of the evidence and what is relevant to your own research question) and finally to the use of the literature to authorise the arguments that you are making.^2, 3^ Therefore, the literature review is a critical synthesis, not simply a description of ‘who said what’. It should be a critical engagement with the established research on your topic. You must read and evaluate critically the strengths and weaknesses of the studies in relation to your research and determine which literature makes a significant contribution to the topic

When constructing your arguments, it may be helpful to first outline the logical flow by the use of headings and subheadings. Each paragraph should establish a key point in your argument and be linked together in a logical sequence. Within the argument it is often helpful to move from the general to the more specific, for example, from discussion of global evidence to regional or national and finally to local.

Try to obtain original or primary sources and do not support a point by citing a reference out of context. Do not copy and paste material from the internet into your writing. To prevent your inadvertently plagiarising material, it is better to make notes from the literature to capture the salient points and then use your notes to write your argument. If you do quote or copy material from other authors then use inverted commas to indicate this and always reference the source.

It is important that you do a spelling and grammar check once your writing is complete. It is also helpful to ask someone else to read through your completed writing to ensure that it flows and is coherent. It is always a good idea to submit your work via Turnitin or another anti-plagiarism software programmes to ensure that you have not inadvertently plagiarised the work of others.

## Using the literature in your proposal

The structure of the proposal has been outlined in the first article of this series.^1^ You will have seen from this article that we do not advocate for a section entitled ‘literature review’ as this does not make the purpose of your writing clear in terms of the proposal. Rather, we have suggested that you have an introduction or background section to your proposal that makes an argument, both consciously and explicitly, for the social and scientific value of your study.

### Making an argument for the social value of your study

The social value speaks to the importance or relevance of the proposed research study in your context. This often includes information about the size or scale of the problem to be addressed, the importance of the issue to improving care and the likely benefits to the healthcare system, patients or society of performing this research study.

For example, in a study on pain in children with HIV, you may argue that HIV and pain are common and important problems in children taking antiretroviral medication in South Africa and that understanding the different types of pain and how to assess them will improve the quality of care for such children. Each of these points should be established by use of the evidence.

### Making an argument for the scientific value of your study

The scientific value speaks to the original contribution that this study will make. This requires a critical appraisal of what is already known about the specific topic or problem and clarification of the knowledge gap that this study will address. The argument is focused on your research question and specific topic and should not involve a lengthy summary of general background information. At a Master's level the originality may often be contextual, in the sense that the study has not been done in the researcher's specific location before. The aim and objectives of the study should usually flow logically from the argument for the scientific value of the study.

### Constructing a conceptual framework

In some research proposals, it is also necessary to describe the theoretical foundation for your study. Sometimes these theories or underlying concepts are linked together into a model of the relationships between them, which is also called a conceptual framework. The conceptual framework provides an explicit framework that helps to order your thinking, provides internal cohesion to your proposal and may guide your methodological choices. This framework is not static and may evolve as you conduct your research. Trafford and Leschem state that:

[*d*]eveloping a conceptual framework forces you to be explicit about what you think you are doing. It also helps you to be selective; to decide which are the important features; which relationships are likely to be of importance or meaning; and hence, what data you are going to collect and analyse.^4^

**FIGURE 1:**
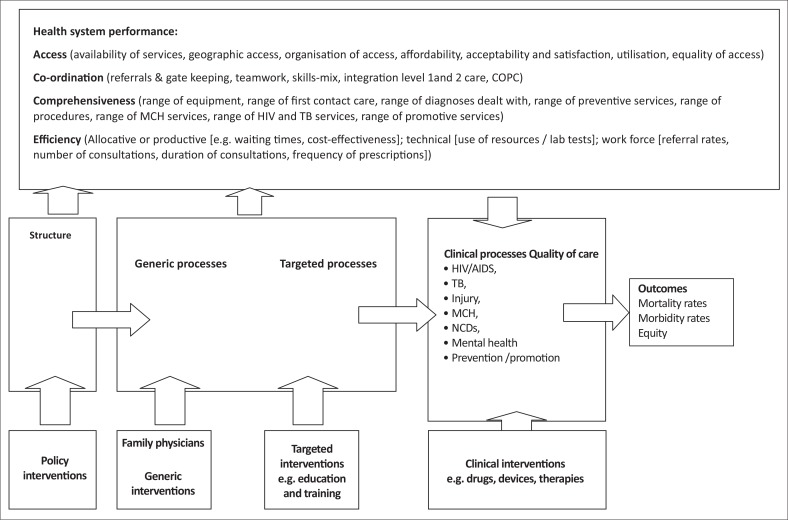
Conceptual framework of the health benefits of employing a Family Physician in the district. COPC, community-oriented primary care; MCH, maternal and child health; TB, tuberculosis; NCDs, non-communicable diseases.

For example, a study on the health benefits of family physicians to the district health services developed the conceptual framework shown in Figure 1 to guide the study design.^5, 6^ The framework shows how family physicians can be considered to be a generic intervention that will potentially impact on health system performance, clinical processes and eventually health outcomes. Any evaluation of their impact should also monitor other policy, targeted and clinical interventions that may also impact on these areas. The model helps decide what to measure and how to conceptually link together the different concepts.

### Informing study design and methods

As you familiarise yourself with the literature, you may also obtain useful guidance on the study design and methods. Think about how other researchers studied this issue and what would be an appropriate way for you to study your research question? You may gain useful insights into methodological problems such as confounding variables, sample selection, calculating sample size, allocation of control groups or statistical analyses. Sometimes you may identify specific tools, such as questionnaires, that could be adapted to your own study. Using validated tools may not only improve the scientific validity of your own study but also enable you to compare your results more easily to other published work.

## Keeping track of information and referencing

After reading an article, make a critical summary of its salient points and how they relate to your research study in terms of helping you with the argument for social value, scientific value, conceptual framework or methodological issues.^3^ Many of these articles will also be useful when you discuss the results of your study later on.

It is critically important to record the full details of each article you have read and summarised – the title of the article, author(s) name(s), source and date of publication – all of which are required when drawing up a reference list. Keeping good records will save you hours of effort and frustration at the end of your literature review. There are a number of software programmes that help you to store the essential information (e.g. Ref Works, Reference Manager or EndNote) and to then automatically cite the references and generate reference lists in different styles. It is a good idea to invest in the time and energy required to learn how to use one of these programmes and to systematically enter the literature you use.

Referencing is an important way of acknowledging where ideas have come from. References must be accurate and complete so as to enable readers to follow up on sources. Reference are made in the text at the relevant place (usually a number or author's surname and date), as well as at the end of the article, where full bibliographic details are provided. All references noted in the text should appear in the reference list and all references in this list must be cited in the text. The two most common ways to reference are:

- Vancouver system (number sequence): Each reference is given a consecutive number in the text and in the reference list at the end of the document there is a list of the full references arranged in *numerical order* (i.e.. the order in which they have been cited in the text).
- Harvard scheme (author-date): Each reference uses the name of the author(s) and date of publication in the text and the reference list is arranged *alphabetically by author*.

Whichever style you choose, it is important, above all, to be consistent in your approach.
